# Cecropin P1 inhibits porcine reproductive and respiratory syndrome virus by blocking attachment

**DOI:** 10.1186/s12866-014-0273-8

**Published:** 2014-11-18

**Authors:** Chunhe Guo, Yumao Huang, Peiqing Cong, Xiaohong Liu, Yaosheng Chen, Zuyong He

**Affiliations:** State Key Laboratory of Biocontrol, School of Life Sciences, Sun Yat-sen University, North Third road, Guangzhou Higher Education Mega Center, Guangzhou, Guangdong 510006 PR China; College of Veterinary Medicine, South China Agricultural University, Guangzhou, Guangdong 510642 PR China

**Keywords:** Cecropin P1, PRRSV, Antiviral activity, Antimicrobial peptide

## Abstract

**Background:**

Porcine reproductive and respiratory syndrome virus (PRRSV) is a continuous threat to the pig industry, causing high economic losses worldwide. Current vaccines have specific limitations in terms of their safety and efficacy, so the development of novel antiviral drugs is urgently required. The aim of this study was to evaluate the inhibitory effects and underlying molecular mechanisms of the antimicrobial peptide cecropin P1 (CP1) against PRRSV infection *in vitro*.

**Results:**

CP1 not only displayed extracellular virucidal activity against PRRSV, but also exerted a potent inhibitory effect when added either before, simultaneously with, or after viral inoculation. The inhibitory effect of CP1 occurred during viral attachment, but not at viral entry into Marc-145 cells. CP1 also inhibited viral particle release and attenuated virus-induced apoptosis during the late phase of infection. CP1 exerted similar inhibitory effects against PRRSV infection in porcine alveolar macrophages, the cells targeted by the virus *in vivo* during its infection of pigs. The expression of interleukin 6 was elevated by CP1 in porcine alveolar macrophages, which might contribute to its inhibition of PRRSV infection.

**Conclusions:**

Collectively, our findings provide a new direction for the development of potential therapeutic drugs against PRRSV infection.

## Background

Porcine reproductive and respiratory syndrome (PRRS) is one of the most economically important viral diseases in sows, causing enormous production losses in the pig industry worldwide [1]. It was first reported at the end of the 1980s in North America and Canada, and is commonly known as blue-eared pig disease [2,3]. The disease is characterized by abortion and poor reproductive performance in pregnant sows and by respiratory distress in growing pigs and piglets [4]. PRRS virus (PRRSV), the etiological agent of the disease, is an enveloped, single-stranded positive-sense RNA virus that clusters in the order *Nidovirales* and the family *Arteriviridae*, together with equine arteritis virus, lactate dehydrogenase-elevating virus, and simian hemorrhagic fever virus. The 15-kb genome of PRRSV contains a 5′-untranslated region (UTR), nine open reading frames (ORFs 1a, 1b, 2a, 2b, and 3–7) and a 3′-UTR [5]. The PRRSV virion consists of a nucleocapsid surrounded by a lipid envelope. Pigs persistently infected with PRRSV develop viremia and reduced cell-mediated immunity [6]. Important features of PRRSV are its extreme genetic, antigenic, and immunobiological variability. Consequently, PRRSV remains the greatest challenge for the swine industry, and there is a strong demand for the development of new antiviral strategies against PRRSV infection [7].

Antimicrobial peptides are cationic and amphipathic molecules distributed widely among organisms of both the plant and animal kingdoms that display broad-spectrum antimicrobial activities against bacteria, fungi, and viruses, thus acting as innate antibiotics [8,9]. Cecropin P1 (CP1), originally isolated from porcine intestine, is a low-molecular-weight peptide with antiviral activity against infectious hematopoietic necrosis virus, viral hemorrhagic septicemia virus, snakehead rhabdovirus, and infectious pancreatic necrosis virus *in vitro* [10,11]. In this study, we investigated whether CP1 inhibits PRRSV replication *in vitro*. Our findings show that CP1 exerted potent antiviral activity against PRRSV infection in both Marc-145 cells and porcine alveolar macrophages (PAMs). The mechanisms of CP1 were also characterized.

## Results

### CP1 markedly inhibits CH-1a infection and replication

**Figure 1:**
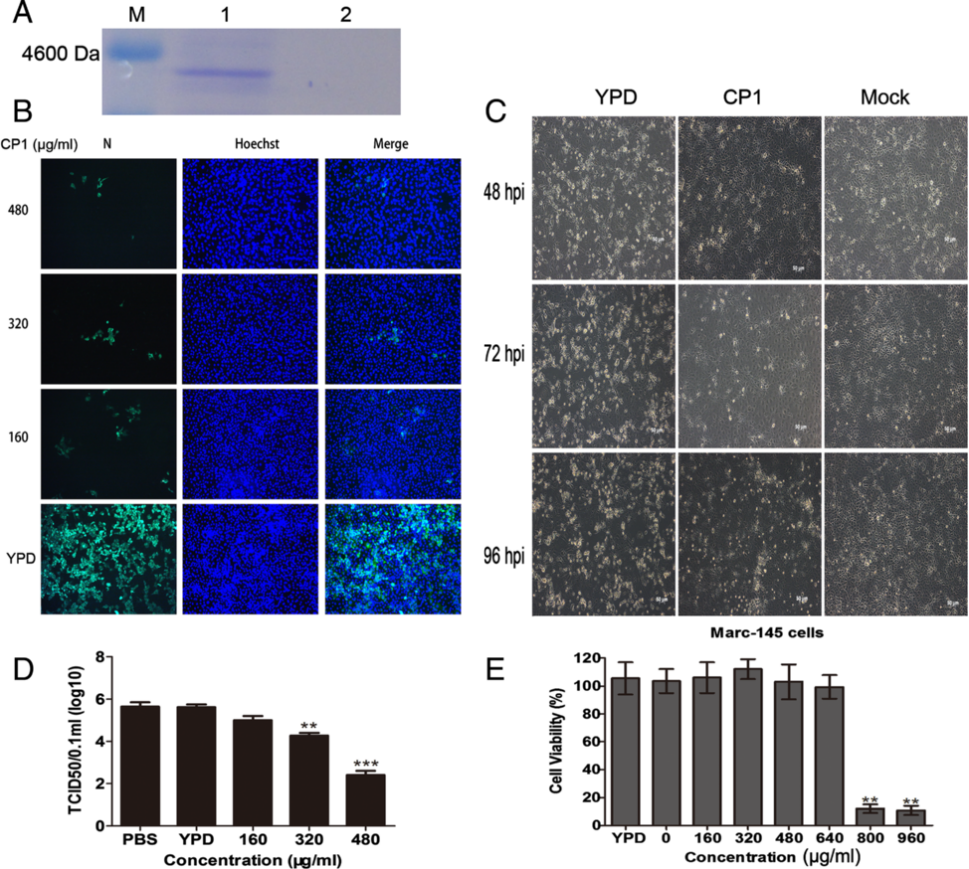
**CP1 strongly inhibits CH-1a infection and replication in Marc-145 cells. (A)** Expression and purification of CP1 in *P. pastoris.* Lane 1 was the purified CP1 (3300 Da). Lane 2 was control. M was protein molecular weight marker. (**B** and **D**) Cells were infected with CH-1a (MOI =0.01) in the presence of different concentrations of CP1, YPD (control), or PBS for 36 h at 37°C. Cells were detected with IFA **(B)**, and the viral yields were detected in the supernatants **(D)**. **(C)** Cells were mock infected or infected with CH-1a (MOI =0.01) in the presence of CP1 (480 μg/ml) or YPD (control). A virus-produced CPE was observed with bright-field microscopy at 48, 72, and 96 hpi. **(E)** Potential cytotoxicity of CP1 against Marc-145 cells was detected with alamarBlue®. A series of concentrations of CP1 or YPD (control) was applied to 60%–70% confluent cells for 48 h, and then 10 μl of alamarBlue® was added for another 3 h. The data are the results of three independent experiments (means ± SE). Significant differences compared with the YPD-treated control group are denoted: *P <0.05, **P <0.01, or ***P <0.001.

CP1 was initially expressed and purified in *Pichia pastoris* (Figure 1A). Subsequently, to evaluate the *in vitro* antiviral activity of CP1 against CH-1a infection, immunofluorescence assay (IFA), cytopathic effect (CPE), and the 50% tissue culture infectious dose (TCID_50_) assays were performed. As shown in Figure 1B, CP1 significantly inhibited viral infection in a dose-dependent manner at 36 h postinfection (hpi). The number of infectious viral particles was markedly reduced by CP1. Consistent with this, CP1 (480 μg/ml) notably abrogated the CH-1a-induced CPE at 48, 72, or 96 hpi in Marc-145 cells infected with CH-1a at a multiplicity of infection (MOI) of 0.01 compared with the yeast extract peptone dextrose (YPD) control (Figure 1C). Furthermore, the production of viral progeny was significantly reduced at 36 hpi by CP1, in a dose-dependent manner (Figure 1D). CP1 inhibited CH-1a infection in Marc-145 cells with a 50% effective concentration (EC_50_) of 112 μg/ml. To rule out the possibility that YPD affects CH-1a replication, cells treated with phosphate-buffered saline (PBS) were used as another control. The results showed that YPD had no effect on viral replication (Figure 1D). To determine whether the concentration-dependent cytotoxicity of CP1 affected CH-1a infection and replication, a cytotoxicity assay was performed in the presence of different concentrations of CP1 or YPD (control) using alamarBlue®. As shown in Figure 1E, no significant cytotoxicity was observed up to 480 μg/ml CP1. Therefore, we performed all subsequent experiments with CP1 at a concentration no higher than 480 μg/ml in Marc-145 cells. The 50% cytotoxic concentration (CC_50_) of CP1 for Marc-145 cells was estimated to be 719 μg/ml.

**Figure 2:**
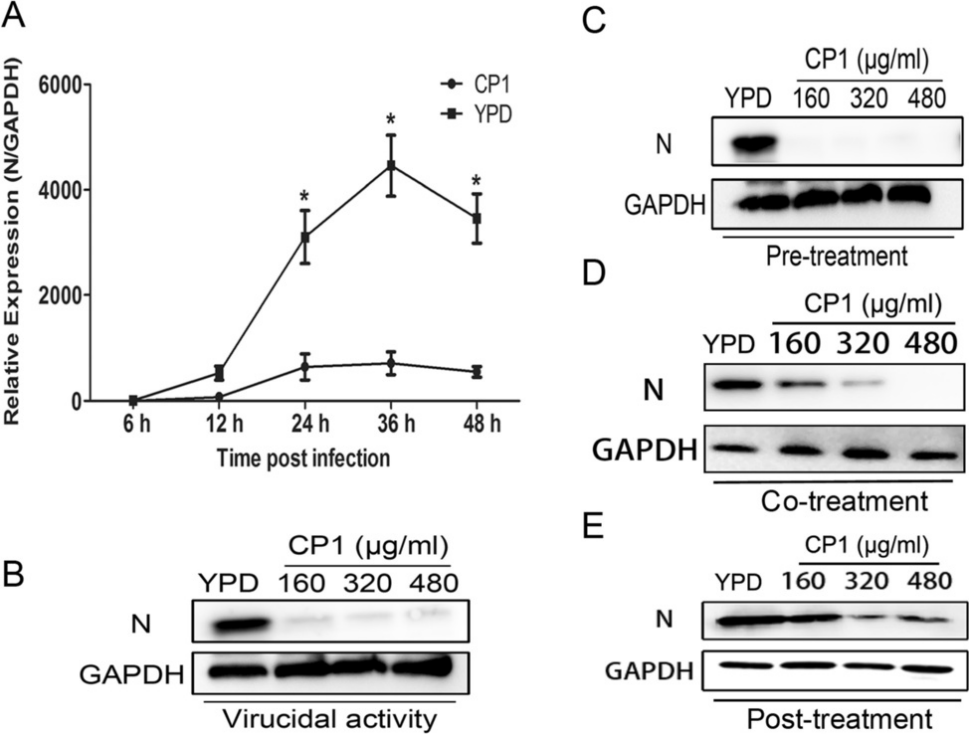
**Viral gene transcription and protein translation were inhibited by CP1 treatment. (A)** Cells were inoculated with CH-1a at an MOI of 0.1 in the presence of CP1 (480 μg/ml) or YPD (control) for 6, 12, 24, 36, or 48 h. Viral RNA transcription was determined with qRT–PCR at different time points after treatment. The level of PRRSV ORF7 mRNA was normalized to the level of GAPDH mRNA in the same sample. **(B)** CP1 or YPD (control) was added to CH-1a for 2 h at 37°C, and the mixture was then used to infect cells for 2 h at 37°C. The inoculum was removed and the cells were cultured for another 36 h and then harvested for immunoblotting analysis. (**C**, **D**, and **E**) CP1 treatment was performed either before (pretreatment) **(C)**, simultaneously with (cotreatment) **(D)**, or after viral infection (posttreatment) **(E)** for 36 h. Viral protein levels were determined with immunoblotting. Data are the results of three independent experiments (means ± SE). Significant differences compared with the YPD-treated control group are denoted: *P <0.05, **P <0.01, or ***P <0.001.

To verify that viral RNA transcription and protein translation are blocked by CP1 during PRRSV infection, the kinetics of CH-1a replication in Marc-145 cells were investigated at the indicated times after treatment with CP1 (480 μg/ml) or YPD (control). Cells were inoculated with CH-1a at an MOI of 0.1 in the presence of CP1 or YPD (control) for 6, 12, 24, 36, or 48 h. Their total RNAs were analyzed with quantitative real-time reverse-transcription polymerase chain reaction (qRT-PCR) to determine the transcript levels of viral ORF7 after treatment. The cells treated with CP1 (480 μg/ml) showed a significant reduction in viral RNA at 24, 36, and 48 hpi compared with those treated with the YPD control (Figure 2A). The effects of treatment with CP1 at the viral protein level were analyzed with three different approaches, as described in the Methods. As shown in Figure 2C–E, compared with the YPD control, the expression of the viral N protein was depressed significantly reduced by CP1 when administered with either the pre-, co-, or posttreatment method. A virucidal assay was performed to investigate the direct inactivation effect of CP1. As shown in Figure 2B, CP1 effectively inactivated the CH-1a virions at 36 hpi. Taken together, these data indicate that CP1 exerts a potent extracellular virucidal activity and an inhibitory effect on CH-1a infection *in vitro*.

### CP1 blocks CH-1a-induced apoptosis during the late phase of infection

**Figure 3:**
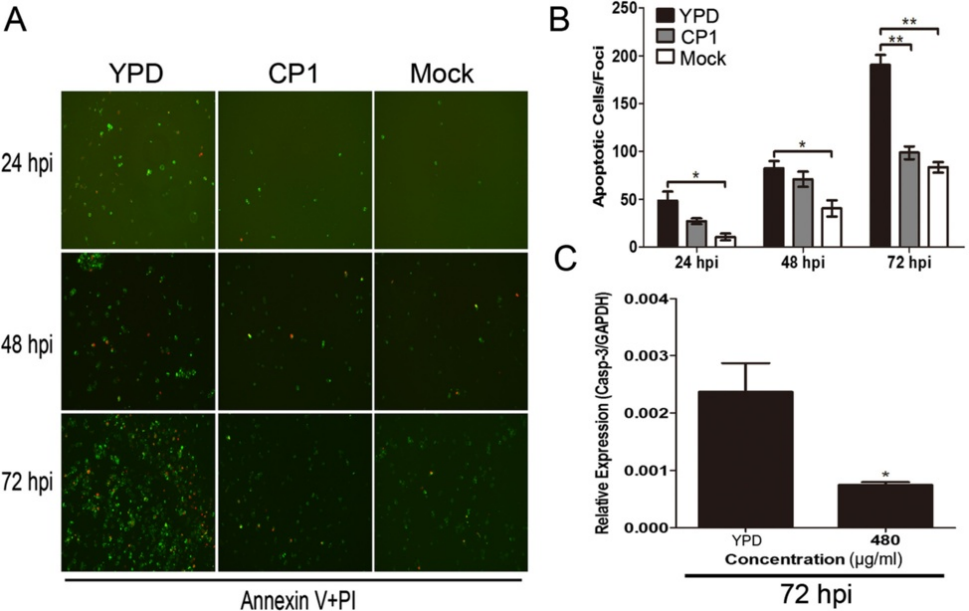
**Effect of CP1 on CH-1a-induced apoptosis during the late phase of infection.** (**A** and **B**) Marc-145 cells were treated with or without CH-1a (MOI =0.01) in the presence of CP1 (480 μg/ml) or YPD (control) for 24, 48, or 72 h, and then stained with FITC–annexin V (green) and PI (red) for 15 min in the dark for the IFA analysis **(A)**. The number of cells calculated from IFA stained with annexin V and PI per foci were counted at the indicated times **(B)**. **(C)** The inhibitory effects of CP1 on apoptosis were confirmed with the transcript levels of caspase-3 in virus-infected cells treated with CP1 for 72 h. Data are the results of three independent experiments (means ± SE). Significant differences compared with the YPD-treated control group are denoted: *P <0.05, **P <0.01, or ***P <0.001.

Previous studies have shown that PRRSV stimulates antiapoptotic pathways in Marc-145 cells and PAMs early in infection, and that PRRSV-infected cells die from apoptosis late in infection [12]. To examine whether CP1 attenuates virus-induced apoptosis during the late phase of infection, an annexin V fluorescein isothiocyanate (FITC)/propidium iodide (PI) assay was performed. CP1 notably reduced the number of apoptotic cells compared with cells treated with the YPD control (Figure 3A and 3B). The inhibitory effects of CP1 on apoptosis were confirmed by the levels of caspase 3 transcripts in the virus-infected cells treated with CP1 for 72 h (Figure 3C). Together, these results indicate that CP1 blocks CH-1a-virus-induced apoptosis during the late phase of infection, which might contribute to the inhibition of PRRSV infection.

### CP1 inhibits viral particle release

**Figure 4:**
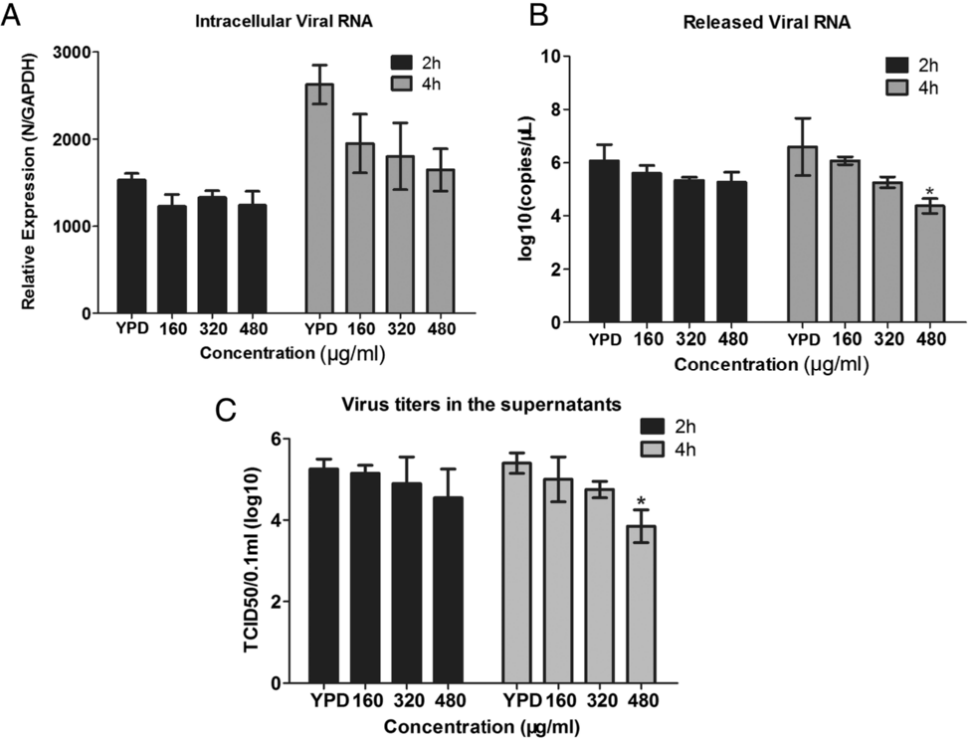
**CP1 blocks CH-1a viral particle release.** (**A** and **B**) Cells were infected with CH-1a at an MOI of 0.01 at 37°C for 24 h, and then treated with different concentrations of CP1 or YPD (control) for 2 or 4 h. The cells and supernatants were harvested to quantify the intracellular **(A)** and extracellular **(B)** viral particles. **(C)** Viral titers in the supernatants were measured after CP1 (480 μg/ml) treatment for 2 or 4 h. Data are the results of three independent experiments (means ± SE). Significant differences compared with the YPD-treated control group are denoted: *P <0.05, **P <0.01, or ***P <0.001.

To investigate whether CP1 reduces viral particle release, an assay described previously [13] was performed to quantify the viral particles in both the cells and the supernatant. Marc-145 cells were treated as described in the Methods. At each time point, the numbers of intracellular particles in cells treated with different concentrations of CP1 were similar to those in cells treated with YPD (control) (Figure 4A). In contrast, significantly fewer infectious viral particles were released into the supernatant by cells treated with CP1 (480 μg/ml) for 4 h than by YPD-treated cells (Figure 4B). The viral titers in the supernatants showed a similar pattern after treatment with CP1 (480 μg/ml) for 4 h (Figure 4C). Taken together, these data indicate that CP1 (480 μg/ml) inhibits CH-1a particle release.

### CP1 blocks viral attachment during the CH-1a life cycle in Marc-145 cells

**Figure 5:**
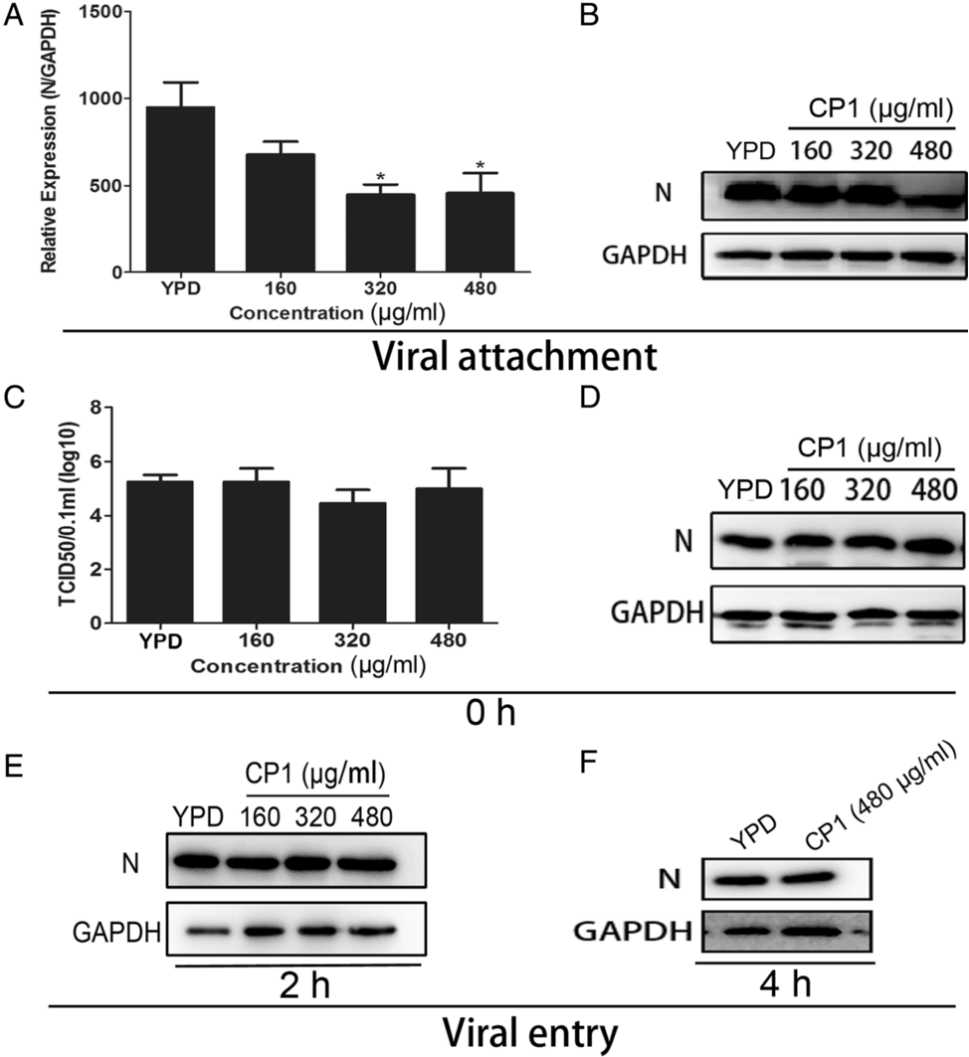
**CP1 blocks viral attachment but not viral entry into Marc-145 cells.** (**A** and **B**) In an attachment assay, cells were cooled at 4°C for 30 min, and then challenged with CH-1a (MOI =0.01) in the presence of various concentrations of CP1 or YPD (control) for 3 h at 4°C. Viral RNA transcription **(A)** and protein levels **(B)** were analyzed. (**C**, **D**, **E**, and **F**) In an entry assay, the kinetics of the antiviral activity of CP1 against CH-1a were evaluated with time-of-addition assays. Cells were challenged with CH-1a (MOI =0.01) for 3 h at 4°C and then incubated at 37°C in the presence of CP1 or YPD (control) for 6 h. CP1 was added at 0, 2, or 4 h (the time point at which the cells were switched to 37°C was set to 0 h). The cells were then rinsed and incubated for another 24 h at 37°C. The inhibitory effects were determined when CP1 was added at 0 (**C** and **D**), 2 **(E)**, of 4 h **(F)** after the cells were shifted to 37°C. Data are the results of three independent experiments (means ± SE). Significant differences compared with the YPD-treated control group are denoted: *P <0.05, **P <0.01, or ***P <0.001.

To characterize the molecular mechanism underlying the antiviral activity of CP1 against CH-1a and to identify the stage of the viral life cycle interrupted by CP1, a viral attachment assay was performed to test whether CP1 blocks CH-1a attachment to Marc-145 cells. CP1 significantly reduced the transcript levels of viral ORF7 in CP1-treated cells during the period of viral attachment (Figure 5A). Consistent with these findings, the N protein levels of CH-1a were blocked by 480 μg/ml CP1 (Figure 5B). These data suggest that CP1 markedly reduces the number of infectious viral particles that attach to the cell membrane.

Previous studies have shown that PRRSV is internalized from the Marc-145 cell surface within 3–6 h [14,15]. Therefore, we investigated whether CP1 also acts at the entry stage of the viral life cycle. The kinetics of the antiviral activity of CP1 against CH-1a were analyzed with time-of-addition assays. CP1 had little effect on the viral titers and protein levels when it was added immediately after the temperature shift (Figure 5C and 5D). Consistent with these findings, no inhibitory effect was observed when CP1 was added at 2 (Figure 5E) or 4 h (Figure 5F) after the cells were shifted to 37°C. Taken together, these data indicate that CP1 blocks the viral attachment process rather than entry process in Marc-145 cells.

### CP1 strongly inhibits CH-1a replication and partially elevates cytokine expression in PAMs

**Figure 6:**
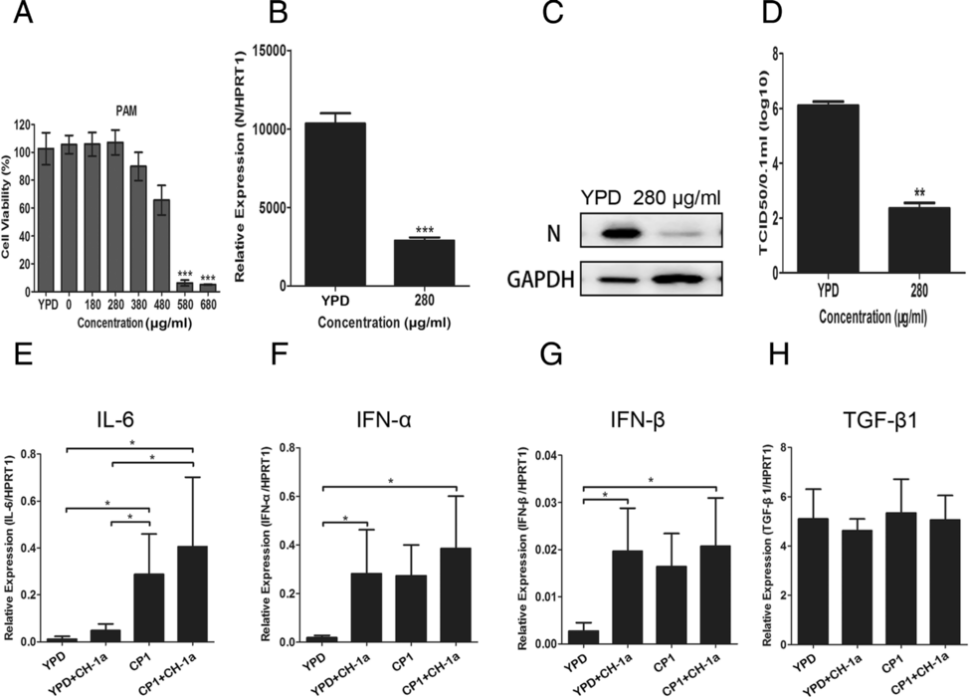
**Antiviral activity and expression of cytokines in PAMs treated with CP1. (A)** A cell viability assay was performed with PAMs and alamarBlue®. (**B**, **C**, and **D**) PAMs were infected with CH-1a (MOI =0.01) in the presence of CP1 or YPD (control) for 36 h at 37°C. Viral RNA transcription **(B)** and protein levels **(C)** and the viral titers in the supernatants **(D)** were analyzed. (**E**, **F**, **G**, and **H**) Virus-infected or uninfected PAMs were cultured in the presence of CP1 (280 μg/ml) or YPD (control) for 18 h. The expression of proinflammatory cytokines IL-6 **(E)**, IFN-α **(F)**, and IFN-β **(G)** and the immunosuppressive cytokine TGF-β1 **(H)** was analyzed. Data are the results of three independent experiments (means ± SE). Significant differences compared with the YPD-treated control group are denoted: *P <0.05, **P <0.01, or ***P <0.001.

Because CP1 effectively inhibited CH-1a infection and replication in Marc-145 cells, we investigated whether CP1 inhibits CH-1a replication in PAMs, the major target cell type of PRRSV infection in pigs *in vivo*. We initially evaluated its effect on CH-1a replication in PAMs. As shown in Figure 6B–D, CP1 caused significant reductions in the viral N protein at the transcript and protein levels and in the viral titers at 36 hpi, compared with those of cells treated with the YPD control. CP1 inhibited CH-1a infection of PAMs with an EC_50_ of 65 μg/ml. To exclude the possibility that CP1 exerts a nonspecific toxicity that affected PAM viability and thus affected CH-1a replication, cytotoxicity assays were performed in the presence of different concentrations of CP1 or YPD (control) for 48 h using alamarBlue®. CP1 was noncytotoxic at concentrations up to 280 μg/ml (Figure 6A). The CC_50_ of CP1 for PAMs was estimated to be 551 μg/ml.

To further characterize the innate immune response induced by CP1 in PAMs, the expression of IL-6, IFN-α, IFN-β, and TGF-β1 was analyzed in the presence of CP1 or YPD (control) in virus-infected or uninfected cells. CP1 significantly enhanced the expression of IL-6 in virus-infected and uninfected cells, which would help to initiate the adaptive immune response. More importantly, the expression of IL-6 was significantly increased in CP1-treated CH-1a-infected cells compared with its expression in cells treated with CH-1a and YPD or YPD alone (Figure 6E). However, CP1 did not significantly induce the expression of IFN-α (Figure 6F), IFN-β (Figure 6G), or TGF-β1 (Figure 6H) in PAMs. Taken together, these data show that CP1 partially induces cytokine expression in PAMs, which might contribute to the inhibition of PRRSV infection.

## Discussion

PRRSV first emerged in the late 1980s in North America, and subsequently in Europe, causing huge economic losses. It has since spread across the globe, inducing characteristic severe reproductive failure in female pigs and respiratory tract illness in piglets [16,17]. PRRSV infection causes the delayed appearance and low titers of neutralizing antibodies, and a weak cell-mediated immune response [18]. This allows the infection to persist and the prevalence of PRRSV infection is still high in the swine industry worldwide. Moreover, current vaccines are losing their power with the antigenic variability and genetic drift of the virus [19,20]. Therefore, there is an urgent need for safer and more effective strategies to control PRRSV. CP1 is a 31-residue peptide, originally isolated from porcine intestine, with a basic N-terminus connected to a neutral C-terminus by a glycine–proline link [21,22]. It displays antibacterial and antiviral properties in fish species important to aquaculture [11]. To our knowledge, this is the first study to report the extracellular virucidal activity and inhibitory effects of CP1 against PRRSV infection and replication *in vitro*, with no cytotoxicity (Figures 1, 2, and 6). CP1 inhibited PRRSV infection with an EC_50_ of 112 μg/ml in Marc-145 cells and 65 μg/ml in PAMs. CP1 also displays antibacterial activity against *Escherichia coli* strains DH5α, TOP10, and K88, *Staphylococcus aureus* strain Cowan I, and *Streptococcus zooepidemicus* strain C55138 (data not shown).

In this study, we have shown that CP1 potently blocked N-PRRSV strain CH-1a replication at multiple points in the viral life cycle, impairing RNA and protein synthesis and viral particle release in Marc-145 cells (Figures 2 and 4). More importantly, an antiviral assay based on three different methods of administration showed that pre- and cotreatments with CP1 (Figure 2C and 2D) produced robust antiviral activity and were more effective against CH-1a infection than CP1 posttreatment (Figure 2E), which is consistent with previous research [23]. This suggests that CP1 has potent inhibitory activity against the initial stages of viral infection, including the viral attachment step, or directly destroys the viral particles. Therefore, we investigated the direct inactivation of the virus by CP1 and found that CP1 has a potent virucidal activity against CH-1a infection *in vitro* (Figure 2B). Further research should be undertaken to clarify the molecular mechanism of the virucidal activity of CP1.

Previously studies have shown that antimicrobial peptides, such as θ-defensin, bind to the glycoprotein B protein of herpes simplex virus type 1 (HSV-1) with high affinity and then protect cells from HSV-1 infection by blocking viral attachment and entry [24]. In this study, we identified the molecular mechanisms underlying the CP1-mediated inhibition of CH-1a *in vitro*. Binding and entry assays in Marc-145 cells showed that CP1 prevents viral adsorption during the viral life cycle (Figure 5), thus disrupting the initial step of viral entry into the target cells, and suggesting that the interaction between the virus and its receptors on the cell membrane is blocked by CP1. However, further studies are required to determine how CP1 affects the attachment of viral particles to the cell membrane. Because Marc-145 cells are not of porcine origin but are monkey cells [25], we also tested the antiviral activity of CP1 in PAMs, which are known to be the primary host cell target for PRRSV replication *in vivo*. CP1 exhibited robust antiviral activity in PAMs (Figure 6B–D), suggesting that it might be an effective inhibitor of PRRSV infection *in vivo*. CP1 also partially elevated cytokine expression (IL-6), which should induce the innate immune response in PAMs (Figure 6E).

## Conclusions

In summary, our findings reveal for the first time that CP1 not only has extracellular virucidal activity, but also exerts a potent inhibitory effect on PRRSV infection and replication *in vitro*, when administered as a pre-, co-, or posttreatment. Therefore, CP1 is an excellent candidate for the development of future antiviral strategies against PRRSV infection. Further research is required to evaluate the potential *in vivo* antiviral activity of CP1 in animal models.

## Methods

### Cells and viruses

Marc-145 cells were grown in Dulbecco’s modified Eagle’s medium (DMEM) with 10% heat-inactivated fetal bovine serum (FBS; PAA, Pasching, Austria). PAMs were obtained with lung lavage from the lungs of 3–8-week-old PRRSV-negative piglets [26] and cultured in RPMI-1640 supplemented with 10% FBS, 100 U/ml penicillin, and 100 μg/ml streptomycin sulfate at 37°C in 5% CO2. All animal experiments were approved by the Institutional Animal Care and Use Committee of Sun Yat-sen University. Classical North American type PRRSV (N-PRRSV) strain CH-1a, kindly provided by Dr. Guihong Zhang of South China Agricultural University, was propagated and titered in Marc-145 cells or PAMs and used throughout the study.

### Expression and purification of CP1 in *Pichia pastoris*

The CP1 gene (GenBank: AB186032.1) was cloned into the expression vector pGAPZaA (Invitrogen) and expressed in *Pichia pastoris* strain SMD1168 (Invitrogen) using the Easyselect™ Pichia Expression Kit (Invitrogen), according to the manufacturer’s instructions. Briefly, the recombinant plasmid pGAPZaA-CP1, confirmed by restriction enzyme digestion, PCR, and sequencing, was transformed into strain SMD1168 with electroporation. The CP1 polypeptide was expressed from the highest-expressing *Pichia pastoris* clone in 10 mL of yeast extract peptone dextrose (YPD) at 30°C with shaking at 250 rpm until the optical density at 600 nm (OD_600_) was 2. An aliquot (0.1 ml) of this culture was used to inoculate 50 ml of YPD in a 250 ml flask, which was then incubated at 30°C with shaking at 250 rpm. The 6 × His-tagged target protein was purified with Ni-NTA agarose (Invitrogen), according to the manufacturer’s protocol. The concentration of the purified recombinant CP1 was determined relative to a bovine serum albumin (BSA) standard using the BCA Protein Assay Kit (Pierce). The purified protein was stored in YPD.

### Cytotoxicity assay

The potential cytotoxicity of CP1 was measured with the alamarBlue® Assay (Invitrogen), according to the manufacturer’s instructions. Briefly, a series of concentrations of CP1, diluted in DMEM or YPD (control), was applied to 60%–70% confluent Marc-145 cells or PAMs. After incubation for 48 h at 37°C in a 5% CO_2_ atmosphere, 10 μl of alamarBlue® was added and the cultures were incubated for another 3 h. Fluorescence intensity was measured at 570 nm excitation and 590 nm emission wavelengths and compared with the control values. The CC_50_ was analyzed with the GraphPad Prism software (version 5.0).

### Quantitative real-time reverse-transcription polymerase chain reaction (qRT-PCR)

Total RNA was extracted from Marc-145 cells or PAMs with TRIzol Reagent (Invitrogen), according to the manufacturer’s instructions, and subjected to qRT-PCR analysis. The reverse transcription of RNA and qPCR were conducted as previously described [27,28]. All samples were run in triplicate. Serial 10-fold dilutions of the CH-1a ORF7 fragment, which encodes the viral N protein, were used to construct a standard curve. Specific primers for the quantitative analysis of mRNAs were designed and are listed in Table 1.

**Table 1:** **List of primers for real-time PCR**

**Primer** ^**#**^	**Sequences (5′-3′)**
ORF7(N)-F	AAAACCAGTCCAGAGGCAAG
ORF7(N)-R	CGGATCAGACGCACAGTATG
mGAPDH-F	TGACAACAGCCTCAAGATCG
mGAPDH-R	GTCTTCTGGGTGGCAGT GAT
mCaspase-3-F	ATGTCCGGGATCTGGGTTCT
mCaspase-3-R	CTAGGTCAAGCTTTCATTCT
pHPRT1-F	TGGAAAGAATGTCTTGATTGTTGAAG
pHPRT1-R	ATCTTTGGATTATGCTGCTTGACC
pIL-6-F	AATGTCGAGGCTGTGCAGATT
pIL-6-R	TGGTGGCTTTGTCTGGATTCT
pTGF-β1-F	AGGGCTACCATGCCAATTTCT
pTGF-β1-R	CCGGGTTGTGCTGGTTGT
pIFN-α-F	TCCAGCTCTTCAGCACAGAG
pIFN-α-R	AGCTGCTGATCCAGTCCAGT
pIFN-β-F	GGAGACAATCCTGGAGGAAAT
pIFN-β-R	TTCAGGTGAAGAATGGTCATGT

^#^F: forward primer, R: reverse primer. The “m” prior to a primer name indicates it is for a monkey gene, and the “p” prior to a primer name indicates it is for a porcine gene.

### Immunoblotting

Marc-145 cells or PAMs were seeded in six-well plates and harvested in lysis buffer containing 1 mM phenylmethylsulfonyl fluoride (Beyotime), and an immunoblotting analysis was performed as previously described [29]. The cell lysates were separated with 10% sodium dodecyl sulfate polyacrylamide gel electrophoresis, and then electroblotted onto pre-equilibrated polyvinylidene difluoride membranes (Millipore). After the membranes were blocked with 5% nonfat dry milk in TBST (20 mM Tris [pH 7.5], 150 mM NaCl, 0.5% Tween 20) for 2 h at 37°C, they were rinsed and incubated with an anti-PRRSV N protein monoclonal antibody (SDOW17), anti-glyceraldehyde phosphate dehydrogenase (GAPDH) antibody (Cell Signaling Technology), horseradish-peroxidase-conjugated anti-mouse IgG antibody (Santa Cruz), and anti-rabbit IgG antibody (Cell Signaling Technology). The signals were detected using ECL reagent (Pierce, Rockford, USA).

### Immunofluorescence assay (IFA)

The cells were fixed with 4% paraformaldehyde and permeabilized with 0.3% Triton X-100 at room temperature, and then blocked with 1% BSA in PBS, after which they were rinsed three times with PBS. After incubation for 30 min, anti-PRRSV N protein mAb (SDOW17) and FITC-conjugated anti-mouse IgG secondary antibody in PBS containing 1% BSA were added sequentially and incubated for another 2 h after each addition. The nuclei were stained with Hoechst dye 33258 (Sigma-Aldrich, St. Louis, MO, USA). The cells were rinsed and observed with fluorescence microscopy (Carl Zeiss, Jena, Germany).

### Virucidal assay

CP1 at the indicated concentrations or YPD (control) was preincubated with CH-1a for 2 h at 37°C. The mixtures were then diluted and added to 60%–70% confluent Marc-145 cells. After incubation for 2 h at 37°C, the mixtures were removed. Cells were replenished with fresh DMEM containing 2% FBS and cultured for another 36 h at 37°C, and then harvested for immunoblotting analysis.

### Antiviral assay

Marc-145 cells were seeded in six-well plates and grown to 60%–70% confluence at 37°C in 5% CO_2_. The effects of treatment with CP1 were analyzed with three different approaches. (I) Pretreatment: cells were treated with CP1 or YPD (control) for 2 h and then infected with CH-1a (MOI =0.01) for 36 h. (II) Cotreatment: cells were inoculated simultaneously with CH-1a (MOI =0.01) and CP1 or YPD (control) for 36 h. (III) Posttreatment: cells were inoculated with CH-1a (MOI =0.01) for 8 h at 37°C, and then the viral inoculum was removed and fresh DMEM medium supplemented with 2% FBS and containing various concentrations of CP1 or YPD (control) was added. The cells were then incubated for a further 36 h. The supernatants were collected to titrate the viral yields, and the viral titers were determined as TCID_50_ [27]. The cells were harvested for IFA, qRT-PCR, and immunoblotting analysis. The concentration that reduced the CPE by 50% relative to the viral control was estimated with the GraphPad Prism software (version 5.0), and was defined as EC_50_.

### Apoptosis assay

Apoptosis was evaluated with an annexin V-FITC/PI assay (BD Biosciences Pharmingen). Marc-145 cells were challenged with CH-1a (MOI =0.01) in the presence of CP1 (480 μg/ml) or YPD (control) for 24, 48, or 72 h. Apoptosis was then measured with annexin V-FITC/PI staining, according to the manufacturer’s protocol. The cells were then analyzed with fluorescence microscopy (Carl Zeiss).

### Viral release assay

Marc-145 cells were seeded in six-well plates, grown to 60%–70% confluence, and infected with CH-1a at an MOI of 0.01 at 37°C for 24 h. The cells were then treated with different concentrations of CP1 or YPD (control), and washed three times with PBS. After incubation for 2 or 4 h, the supernatants and cells were harvested to quantify the extracellular and intracellular viral particles, respectively, with qRT-PCR.

### Binding and entry assays

Binding and entry assays were performed as previously described, with modifications [30,31]. For the binding assay, Marc-145 cells were cooled at 4°C for 30 min, and then challenged with CH-1a (MOI =0.01) in the presence of various concentrations of CP1 or YPD (control) for 3 h at 4°C. After the cells were rinsed three times with ice-cold PBS, they were incubated at 37°C for another 24 h. The cell lysates were prepared for qRT-PCR and immunoblotting analysis.

For the entry assay, Marc-145 cells were initially challenged with CH-1a (MOI =0.01) for 3 h at 4°C. After the cells were rinsed three times with ice-cold PBS, they were cultured at 37°C in the presence of various concentrations of CP1 or YPD (control) for 6 h. CP1 was added at 0, 2, or 4 h after the temperature shift (the time point at which the cells were switched to 37°C was set to 0 h). The cells were then rinsed three times with PBS and incubated for another 24 h at 37°C. The supernatants were harvested for virus titration and the cells were collected for immunoblotting analysis.

### Cytokine mRNA expression

The expression of cytokines in PAMs was determined. Virus-infected or uninfected cells were cultured in the presence of CP1 (280 μg/ml) or YPD (control) for 18 h, and the expression of proinflammatory cytokines interleukin 6 (IL-6), interferon α (IFN-α), and IFN-β, and the immunosuppressive cytokine transforming growth factor β1 (TGF-β1) was then quantified with qRT-PCR. Specific primers designed for the quantitative analysis of the mRNAs are listed in Table 1.

### Statistical analysis

All experiments were performed with at least three independent replicates. Data were analyzed with the SPSS 16.0 and GraphPad Prism software (version 5.0) and are expressed as means ± standard errors (SE). Student’s *t* test and one-way analysis of variance were used. *P* values <0.05 were considered statistically significant.

## References

[1] Van Breedam W, Van Gorp H, Zhang JQ, Crocker PR, Delputte PL, Nauwynck HJ (2010). The M/GP(5) glycoprotein complex of porcine reproductive and respiratory syndrome virus binds the sialoadhesin receptor in a sialic acid-dependent manner. PLoS Pathog.

[2] Wensvoort G, Terpstra C, Pol JM, ter Laak EA, Bloemraad M, de Kluyver EP, Kragten C, van Buiten L, den Besten A, Wagenaar F, Broekhuijsen JM, Moonen PL, Zetstra T, de Boer EA, Tibben HJ, de Jong MF, van’t Veld P, Greenland GJ, van Gennep JA, Voets MT, Verheijden JH, Braamskamp J (1991). Mystery swine disease in The Netherlands: the isolation of Lelystad virus. Vet Q.

[3] Zhao C, Liu S, Li C, Yang L, Zu Y (2014). In vitro evaluation of the antiviral activity of the synthetic epigallocatechin gallate analog-epigallocatechin gallate (EGCG) palmitate against porcine reproductive and respiratory syndrome virus. Viruses.

[4] Kuzemtseva L, de la Torre E, Martin G, Soldevila F, Ait-Ali T, Mateu E, Darwich L (2014). Regulation of toll-like receptors 3, 7 and 9 in porcine alveolar macrophages by different genotype 1 strains of porcine reproductive and respiratory syndrome virus. Vet Immunol Immunopathol.

[5] Johnson CR, Griggs TF, Gnanandarajah J, Murtaugh MP (2011). Novel structural protein in porcine reproductive and respiratory syndrome virus encoded by an alternative ORF5 present in all arteriviruses. J Gen Virol.

[6] Hu J, Ni Y, Dryman BA, Meng XJ, Zhang C (2012). Immunogenicity study of plant-made oral subunit vaccine against porcine reproductive and respiratory syndrome virus (PRRSV). Vaccine.

[7] Wang L, Xiao S, Gao J, Liu M, Zhang X, Li M, Zhao G, Mo D, Liu X, Chen Y (2014). Inhibition of replication of porcine reproductive and respiratory syndrome virus by hemin is highly dependent on heme oxygenase-1, but independent of iron in MARC-145 cells. Antiviral Res.

[8] Park CH, Valore EV, Waring AJ, Ganz T (2001). Hepcidin, a urinary antimicrobial peptide synthesized in the liver. J Biol Chem.

[9] Peters BM, Shirtliff ME, Jabra-Rizk MA (2010). Antimicrobial peptides: primeval molecules or future drugs?. PLoS Pathog.

[10] Chiou PP, Lin CM, Perez L, Chen TT (2002). Effect of cecropin B and a synthetic analogue on propagation of fish viruses in vitro. Mar Biotechnol (NY).

[11] Chiou PP, Chen MJ, Lin CM, Khoo J, Larson J, Holt R, Leong JA, Thorgarrd G, Chen TT (2014). Production of homozygous transgenic rainbow trout with enhanced disease resistance. Mar Biotechnol (NY).

[12] Costers S, Lefebvre DJ, Delputte PL, Nauwynck HJ (2008). Porcine reproductive and respiratory syndrome virus modulates apoptosis during replication in alveolar macrophages. Arch Virol.

[13] Kumar N, Liang YH, Parslow TG, Liang YY (2011). Receptor tyrosine kinase inhibitors block multiple steps of influenza a virus replication. J Virol.

[14] Jourdan SS, Osorio FA, Hiscox JA (2012). Biophysical characterisation of the nucleocapsid protein from a highly pathogenic porcine reproductive and respiratory syndrome virus strain. Biochem Biophys Res Commun.

[15] Music N, Gagnon CA (2010). The role of porcine reproductive and respiratory syndrome (PRRS) virus structural and non-structural proteins in virus pathogenesis. Anim Health Res Rev.

[16] Pejsak Z, Stadejek T, Markowska-Daniel I (1997). Clinical signs and economic losses caused by porcine reproductive and respiratory syndrome virus in a large breeding farm. Vet Microbiol.

[17] Neumann EJ, Kliebenstein JB, Johnson CD, Mabry JW, Bush EJ, Seitzinger AH, Green AL, Zimmerman JJ (2005). Assessment of the economic impact of porcine reproductive and respiratory syndrome on swine production in the United States. J Am Vet Med Assoc.

[18] Xiao ZG, Batista L, Dee S, Halbur P, Murtaugh MP (2004). The level of virus-specific T-cell and macrophage recruitment in porcine reproductive and respiratory syndrome virus infection in pigs is independent of virus load. J Virol.

[19] Murtaugh MP, Xiao ZG, Zuckermann F (2002). Immunological responses of swine to porcine reproductive and respiratory syndrome virus infection. Viral Immunol.

[20] Thanawongnuwech R, Suradhat S (2010). Taming PRRSV: revisiting the control strategies and vaccine design. Virus Res.

[21] Moore AJ, Beazley WD, Bibby MC, Devine DA (1996). Antimicrobial activity of cecropins. J Antimicrob Chemother.

[22] Zhou YL, Peng Y (2013). Synergistic effect of clinically used antibiotics and peptide antibiotics against gram-positive and gram-negative bacteria. Exp Ther Med.

[23] Hoffmann J, Schneider C, Heinbockel L, Brandenburg K, Reimer R, Gabriel G (2014). A new class of synthetic anti-lipopolysaccharide peptides inhibits influenza A virus replication by blocking cellular attachment. Antiviral Res.

[24] Yasin B, Wang W, Pang M, Cheshenko N, Hong T, Waring AJ, Herold BC, Wagar EA, Lehrer RI (2004). Theta defensins protect cells from infection by herpes simplex virus by inhibiting viral adhesion and entry. J Virol.

[25] Zhang L, Liu J, Bai J, Du Y, Wang X, Liu X, Jiang P (2013). Poly(I:C) inhibits porcine reproductive and respiratory syndrome virus replication in MARC-145 cells via activation of IFIT3. Antiviral Res.

[26] Qiao SL, Feng LL, Bao DK, Guo JQ, Wan B, Xiao ZJ, Yang SZ, Zhang GP (2011). Porcine reproductive and respiratory syndrome virus and bacterial endotoxin act in synergy to amplify the inflammatory response of infected macrophages. Vet Microbiol.

[27] Delogu I, Pastorino B, Baronti C, Nougairede A, Bonnet E, de Lamballerie X (2011). In vitro antiviral activity of arbidol against Chikungunya virus and characteristics of a selected resistant mutant. Antiviral Res.

[28] Patel D, Stein DA, Zhang YJ (2009). Morpholino oligomer-mediated protection of porcine pulmonary alveolar macrophages from arterivirus-induced cell death. Antivir Ther.

[29] Hudjetz B, Gabriel G (2012). Human-like PB2 627 K influenza virus polymerase activity is regulated by importin-alpha1 and -alpha7. PLoS Pathog.

[30] Yang Q, Gao L, Si J, Sun Y, Liu J, Cao L, Feng WH (2013). Inhibition of porcine reproductive and respiratory syndrome virus replication by flavaspidic acid AB. Antiviral Res.

[31] Hong W, Li T, Song Y, Zhang R, Zeng Z, Han S, Zhang X, Wu Y, Li W, Cao Z (2014). Inhibitory activity and mechanism of two scorpion venom peptides against herpes simplex virus type 1. Antiviral Res.

